# Refining Nutritional Assessment Methods for Older Adults: A Pilot Study on Sicilian Long-Living Individuals

**DOI:** 10.3390/nu17111873

**Published:** 2025-05-30

**Authors:** Anna Aiello, Anna Calabrò, Rosa Zarcone, Calogero Caruso, Giuseppina Candore, Giulia Accardi

**Affiliations:** Laboratory of Immunopathology and Immunosenescence, Department of Biomedicine, Neuroscience and Advanced Diagnostics, University of Palermo, 90133 Palermo, Italy; anna.aiello@unipa.it (A.A.); anna.calabro@unipa.it (A.C.); rosa.zarcone@unipa.it (R.Z.); calogero.caruso@unipa.it (C.C.); giulia.accardi@unipa.it (G.A.)

**Keywords:** aging, health-related nutritional risk, LLIs, nutritional tools, older adults

## Abstract

**Background:** Assessing nutrition-related health risks in older individuals is often overlooked in clinical practice due to the lack of appropriate methods of evaluation. While anthropometric measurements and body composition analyses are mainly used, these tools are not standardized for the oldest old and fail to account for age-related changes. This underscores the need for improved assessment techniques that accurately capture the progressive and non-linear shifts in nutritional status throughout the aging process. Accordingly, the primary aim of our paper is to identify the most effective tools to use for evaluating nutritional status in the oldest population. **Methods:** To address this gap, we conducted a cross-sectional study, investigating the nutritional status of a cohort of Sicilian individuals aged between 65 and 111, using methods commonly applied to adult and older adult populations. These included the BIoimpedance Analysis (BIA), the Mini Nutritional Assessment (MNA) evaluation, and nutritional risk indices such as the COntrolling NUTritional Status (CONUT) score and Geriatric Nutritional Risk Index (GNRI). **Results:** Despite the oldest population being classified as “at risk” of malnutrition by the MNA or “cachetic” by BIA, our results indicated a “normal” or “low risk” of malnutrition when assessments were performed using tools (GNRI and CONUT) that were not reliant on body composition parameters. These findings align with clinical history assessments conducted during their recruitment. **Conclusions:** This pilot study highlights the need for future research aimed at developing standardized, multidimensional assessment models tailored to the heterogeneity of each age group, to improve risk stratification, clinical outcomes, and personalized nutritional care.

## 1. Introduction

Aging is an unavoidable process but aging successfully, mitigating the onset of age-related diseases (ARDs), is achievable. In this regard, numerous studies provide strong and growing evidence that well-balanced diets play a key role in promoting healthy aging and supporting better aging outcomes [1,2,3,4]. These nutritional patterns are based on the so-called “slow-aging diets”, dietary regimens designed to delay the onset of ARDs and slow biological aging, typically by prioritizing plant-based foods, reducing calories and saturated fats, and increasing intake of whole grains, legumes, fruits, and vegetables. These, combined with an active lifestyle, including regular physical activity and reduced sedentary behavior, are essential for preserving both functional and cognitive health over time [5,6]. Conversely, unbalanced nutrition is associated with adverse outcomes such as frailty, sarcopenia, and increased risk of chronic diseases [7].

The relevance of nutritional status in aging becomes even more significant when considered in the context of demographic shifts. The population-aging phenomenon is accelerating in Europe, with individuals aged 65 and over representing 21.6% of the total population (data updated to 1 January 2024), with an estimated rise to 32.5% by 2100. Italy, in particular, leads the continent in terms of the old-age index, which rose from 161 in 2012 to 187 in 2024 [8]. This aging trend poses critical challenges for healthcare systems, especially when it comes to the management of disease-related malnutrition (DRM). Moreover, the economic impact of DRM in Europe is estimated to be up to EUR 120 billion annually, affecting approximately 20 million individuals [9]. In Italy, the situation is particularly concerning: malnutrition affects over 49% of hospitalized patients, up to 69% of those in long-term care facilities, and between 8% and 20% of community-dwelling older adults [10]. Finally, according to the WHO, 30% to 50% of inpatients across Europe are malnourished, and it was highlighted that early nutritional interventions could significantly reduce both disease burden and healthcare costs [11].

Despite this, accurately determining nutritional status and identifying related health risks in older individuals remains a major challenge for the prevention or treatment of DRM. The issue becomes even more complex when long-living individuals (LLIs), the oldest population, are taken into consideration, as they represent a highly specific population for several reasons, not least their exceptional longevity. Achieving extreme age is often the result of a complex interplay of genetic, environmental, and lifestyle factors which are not yet fully understood, nor easily replicated or controlled in research settings [5,12,13]. It is difficult to apply standard clinical or nutritional assessment tools designed for the younger or general older population to these individuals because of their unique physiological characteristics, such as altered metabolism, atypical body composition, and different inflammatory or immune responses. Their rarity, increased frailty, cognitive impairments and reduced accessibility also make recruitment and data collection more challenging. These factors contribute to a lack of standardized protocols and limit the generalizability of findings, making it essential to develop methodologies tailored to this distinct and underrepresented group.

In this context, the main aim of this cross-sectional pilot study is to identify which methods, among those most commonly used in clinical practice to assess nutritional status, i.e., the Mini Nutritional Assessment (MNA) evaluation [14], BIoimpedance Analysis (BIA) [5,15], the COntrolling NUTritional Status (CONUT) score [16], and the Geriatric Nutritional Risk Index (GNRI) [17], are the most informative in describing nutritional status and health risks in LLIs. In particular, the MNA questionnaire has been validated for use in outpatient settings, hospitals, and nursing homes. It was originally developed to provide a quick and comprehensive tool for evaluating nutritional status in individuals aged ≥ 65 years old (y.o.) [14]. BIA was originally validated for healthy adults, mostly aged between 18 and 50 years [5,15]. Few studies have investigated the use of BIA in clinical practice for individuals aged ≥ 65 y.o. For instance, Barbosa-Silva et al. examined reference values up to 95 years of age, and Saragat et al. included individuals up to 100 years [18,19]. However, no formal validation of the method exists for the oldest age group, primarily due to the well-known age-related changes in body composition, such as shifts in hydration, fat redistribution, and sarcopenia, which make it particularly challenging to standardize and validate BIA parameters in this population [5]. Finally, the GNRI was designed for hospitalized older adults and validated in individuals aged ≥ 65 y.o. [17]. It provides information correlated with a severity score that reflects the impact of nutritional status on clinical complications, allowing for the classification of patients according to their risk of morbidity and mortality. Indeed, the GNRI has proven to be a more reliable prognostic indicator of adverse outcomes in elderly inpatients than other indices based solely on albumin or Body Mass Index (BMI) [17]. In contrast, the CONUT score has not yet been formally validated for the older population; however, it was originally developed for the early detection and ongoing monitoring of hospital-related undernutrition and has been widely applied in clinical settings for this purpose [16].

Additionally, we provide a comprehensive overview of the nutritional status and nutrition-related health risks within our recruited cohorts of the Sicilian population, from adults to the oldest old, using a multidimensional approach.

## 2. Materials and Methods

### 2.1. Study Design

This study was reported in accordance with “Strengthening the Reporting of Observational Studies in Epidemiology” (STROBE) guidelines for cross-sectional studies.

All participants or their legal representatives provided written informed consent before enrolment. To ensure confidentiality, each participant was identified with an alphanumeric code, and all data were stored securely in a database that was accessible only to researchers involved in the project.

The recruitment and the sample collection were carried out for the youngest participants (from 65 to 80 y.o.) at Palermo University Hospital. Home visits were conducted for individuals aged between 81 and 111 y.o.

Overnight fasting blood samples were collected in EDTA or dry tubes, and aliquots of blood, plasma, and serum were obtained after centrifugation. Hematological and biochemical analyses, previously described by Aiello et al. [13], were performed to assess the health status of the participants and to calculate the CONUT and GNRI scores.

The study protocol was conducted in accordance with the Declaration of Helsinki and its amendments and was approved by the Ethics Committee of Palermo University Hospital (Nutrition and Longevity, No. 032017).

### 2.2. Participants

A total of 80 subjects (48 females and 32 males, mean age ± standard deviation (SD): 86.9 ± 14.8), aged between 65 and 111 years and free from invalidating diseases, were selected from a larger cohort of 230 individuals enrolled in the project “Discovery of molecular and genetic/epigenetic signatures underlying resistance to ARDs and comorbidities (DESIGN, 20157ATSLF)”, funded by the Italian Ministry of Education, University and Research from June 2017 to 2020.

#### Variables

The subjects were recruited from western and southwestern Sicily and were subdivided into three different age groups: older adults aged between 65 and 80 (N = 33; mean age ± SD = 70.73 ± 4.32); older adults aged between 81 and 94 (N = 13; mean age ± SD = 90 ± 4.48); and the oldest population, named LLIs, i.e., people aged between 95 and 111 (N = 34; mean age ± SD = 101.32 ± 4.06). The three age groups were chosen to reflect progressive and non-linear changes in nutritional status and body composition, based on both clinical and gerontological considerations observed across the aging process.

A comprehensive, interviewer-administered questionnaire was used to collect information on demographic data (i.e., sex, age, educational level, residence history, household composition, marital status, and general self-rated life satisfaction); cognitive and functional status (i.e., mini-mental state examination (MMSE), activities of daily living, and instrumental activities of daily living); lifestyle habits (i.e., smoking habits, physical activity, sleep quality and duration, and social engagement); health status, drug assumption and family medical history. The nutritional status was evaluated using validated questionnaires (i.e., MNA and PREDIMED).

Based on the results of the MMSE, written informed consent was obtained from participants or their legal representatives. Specifically, individuals scoring between 26 and 30, indicating adequate decisional capacity, signed the informed consent themselves. For those scoring between 18 and 25, consent was provided by a legally authorized representative (usually a family member). In all cases, the study was clearly explained, and whenever possible, the participant’s verbal or non-verbal assent was also documented. Only individuals with severe dementia (MMSE < 18) were excluded. Also, participants with multiple incapacitating conditions, chronic invalidating diseases, and acute illnesses were excluded from the study. Most of the LLIs recruited reside with their families, predominantly with their daughters, while 25% live in nursing homes. Additionally, 69% of them have never smoked, and most lead sedentary lives, with few being bedridden. For the anthropometric measurements, we used different approaches, i.e., the classic parameters (circumferences, height, weight), single-frequency BIA, Bioelectrical Impedance Vector Analysis (BIVA), MNA, GNRI, and CONUT scores.

### 2.3. Anthropometric, BIA, and BIVA Evaluation

Anthropometric measurements, including body weight and height, were obtained with subjects lightly dressed and barefoot, and BMI was calculated accordingly.

BIA was conducted according to the method described by Aiello et al. [13]. Briefly, a single-frequency (50 kHz, 800 µA) BIA approach, in a supine position, was used. A tetrapolar electrode configuration was applied, with one pair of electrodes on the back of the hand (the injector electrode on the metacarpophalangeal joint of the third finger and the sensor electrode on the radioulnar joint) and another pair on the back of the ipsilateral foot (injector electrode on the metatarsophalangeal joint of the third toe and sensor electrode on the tibiotarsal joint). A portable Akern BIA 101 device was used, which was suitable for home visits. The analysis measured resistance (Ohm), reflecting total body water volume reactance (Ohm), proportional to cell mass. The Phase Angle (PhA), a vector quantity used as an indicator of cell membrane integrity and hydration status, was calculated as the arctangent of the reactance-to-resistance ratio (Rz/Xc) using BodygramPlus 1.1.4.4 software. The principles, applications, advantages, and limitations of single-frequency BIA have been extensively discussed by Foster et al. and Bera [20,21].

BIVA standardizes the Rz and Xc values for height (Rx/H and Xc/H) and plots them on a nomogram. This normalization process ensures that these values are adjusted for height, making them consistent across individuals of varying body sizes. Doing so allows for more accurate body composition assessments, with reference values that are stratified by age and sex to account for physiological differences among different population groups [18,22]. Reference values for age stratification, related to Xc/H, Rx/H and PhA, taken into consideration, are based on those reported by Saragat et al. [18]. For instance, the PhA cutoffs referenced in the text are those listed in Table S1.

### 2.4. MNA Questionnaire

The MNA questionnaire is a simple and quick method used to identify older subjects at risk of malnutrition, associating anthropometric measurements, dietary questions, a global perspective on health status, and a subjective assessment [23,24].

The MNA consists of a total of 18 questions, divided into two sections: screening and global evaluation, according to Vellas et al., adjusted for Italian administration [14]. It covers global assessment (including lifestyle, medication, and mobility), anthropometric measurements (such as BMI, brachial and calf circumferences, and weight loss), and nutritional assessment (including eating habits, ability to self-feed, and perceived health and nutritional status). A total score was calculated by summing the scores of individual questions, which categorizes participants into three nutritional statuses: (i) MNA ≥ 24 points indicates that a person is well nourished; (ii) 17 ≤ MNA ≤ 23.5 points indicates that they are at risk of malnutrition; (iii) MNA < 17 points indicates malnutrition.

### 2.5. CONUT and GNRI Scores

The GNRI, as a derivative of the nutritional risk index, has been determined according to the following formula: GNRI = (1.489 × albumin (g/L)) × (41.7 × (weight/Ideal weight (WLo)). WLo was determined with the Lorentz formula:

Male: WLo = (H (cm) − 100) − ((H (cm) − 150)/4); female: WLo = (H (cm) − 100) − ((H (cm) − 150)/2). If weight/WLo was greater than 1, it was set equal to 1.

Based on the GNRI score, individuals have been categorized into the following three groups: (i) GNRI < 82 points, severe risk of malnutrition; (ii) 82 ≤ GNRI < 92 points, moderate risk of malnutrition; (iii) 92 ≤ GNRI ≤ 98 points, low risk of malnutrition; (iv) GNRI > 98 points, no risk of malnutrition [25].

The CONUT score was calculated by summing the values obtained from albumin, serum cholesterol levels, and lymphocyte count, in accordance with the criteria established by Toyokawa et al. [25]. Each parameter is assigned a score based on predefined thresholds, and the sum of these scores determines the overall CONUT score. A score ranging from 0 to 1 indicates a normal nutritional status. A score between 2 and 4 signifies light malnutrition. A score in the range of 5 to 8 represents moderate malnutrition. A score between 9 and 12 indicates severe malnutrition.

### 2.6. Bias

Efforts were made to minimize bias using standardized procedures for data collection, including the administration of validated questionnaires and calibrated tools for anthropometric and bioimpedance measurements. All interviews and assessments were conducted by trained personnel to reduce interviewer bias. Given the rare nature of LLIs, the study used all eligible and available participants, which may introduce selection bias, but reflects the real-world demographic composition of this population.

### 2.7. Study Size

No formal a priori sample size calculation was performed, as this study aimed to include all available and eligible participants, with particular focus on the rare and understudied population of LLIs (≥95 years). This approach reflects the exploratory nature of the study and the difficulty of recruiting such individuals in sufficient numbers for standard power calculations.

### 2.8. Statistical Analysis

Hotelling’s T^2^ test, along with the calculation of the *p*-value and Mahalanobis’ generalized distance (D), was used to assess significant differences between age groups in the BIVA. To achieve this, the BIVA software (2002) was used to calculate the confidence interval among the age groups identified, considering parameters such as Rz, Xc, H, SD, and correlation coefficient (r), providing a graphical representation of population mean vectors and variability degree [26]. Linear regression analysis was employed to model the relationship between the study variables (i.e., PhA, BMI) and age. One-way ANOVA was conducted to compare variables across age groups. *p*-values < 0.05 were not considered statistically significant. All statistical analyses were performed using GraphPad Prism 9.

## 3. Results

### 3.1. Nutritional Status Evaluation Based on Anthropometric Measures and BIA

The assessment of anthropometric parameters, particularly BMI, indicates that LLIs fall into the normal-weight category (Table 1).

In contrast, among those aged 65 to 80 years, the distribution is relatively balanced among the normal weight, overweight, and obesity categories (Table 1). For the intermediate age group, spanning 81 to 94, most individuals are classified as either normal weight or overweight (Table 1). The mean BMI values indicated a general condition of overweight in the 65–80 and 81–94 age groups (Table 1).

**Figure 1:**
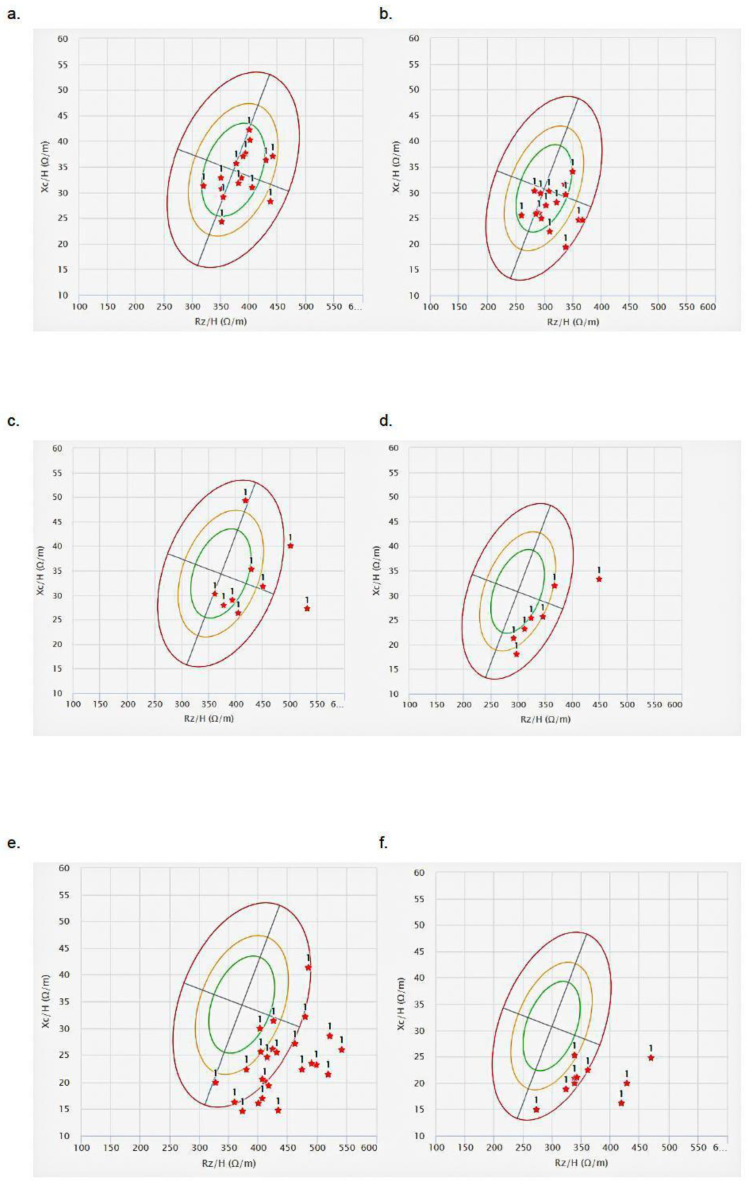
BIA vectors stratified by age and sex. (**a**,**c**,**e**) represent female BIA vectors, while (**b**,**d**,**f**) correspond to male BIA vectors. Panels (**a**,**b**) depict the 65–84 age group, (**c**,**d**) illustrate the 85–94 age group, and (**e**,**f**) represent individuals aged ≥95. Each red star marks the PhA value of an individual.

BIA revealed that PhA distribution shifted downward with advancing age, indicating a progressive decline in hydration and tissue mass, as indicated, taking into consideration the example of confidence intervals reported in Figure S1 compared with Figure 1. Most analyzed individuals, irrespective of age or sex, clustered within the cachectic quadrant (i.e., the lower-right quadrant and outliers).

Most individuals in the 65–80 age group had values near the center of the ellipse, signifying relatively good body composition in terms of hydration and cell mass (Figure 1a,b). Only one individual, belonging to the 81–94 age group, fell into the upper right quadrant, indicative of the athletic type (Figure 1c,d). In contrast, within this group, females seem to have a more favorable body composition compared to males. Notably, in Figure 1e, focusing on the ≥95 age group, most females exhibited outlier values that fell outside the confidence interval (95th percentile), highlighting a pronounced deviation from typical values (i.e., 3–4°, Table S1).

All PhA values, across all age groups, were lower than the normal range established for healthy adults (e.g., ≥6.96°; Table S1).

**Figure 2:**
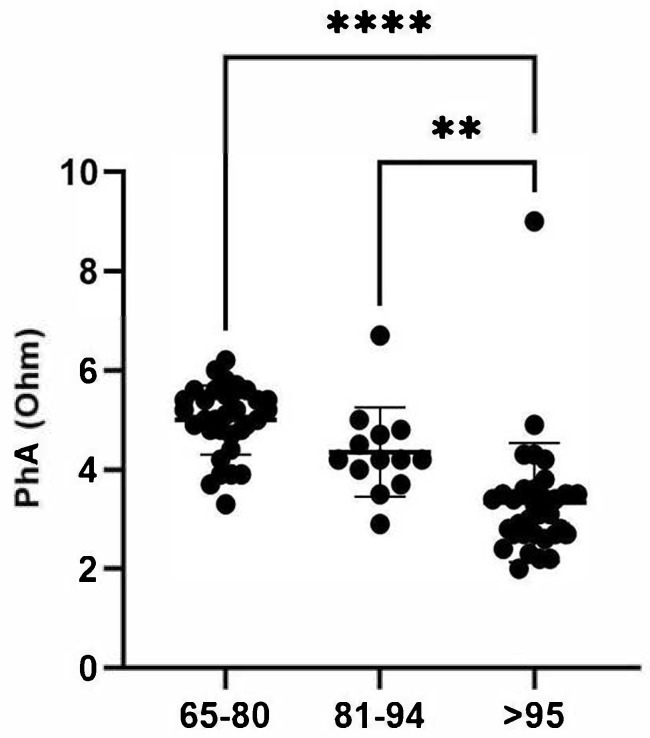
PhA values according to age groups. The scatter plot shows differences between the mean of the values of PhA from each age group obtained by one-way ANOVA. The standard deviation (SD) and *p*-values are shown on the graphs. The vertical lines with horizontal caps represent the mean ± SD. Statistical significance between groups in the columns is denoted by horizontal lines above the bars, marked with asterisks (*). The number of “*” indicates the level of significance: ** *p*-value ≤ 0.01; **** *p*-value ≤ 0.0001. PhA = Phase Angle.

Examining the distribution of PhA values across the different age groups in detail, it is evident that the reduction is statistically significant when comparing the group of LLIs to the group of individuals aged 65–80, with a *p*-value < 0.0001 (Figure 2). However, while the difference between the 81–94 and ≥95 groups is statistically significant, it is less substantial (*p*-value = 0.006).

**Figure 3:**
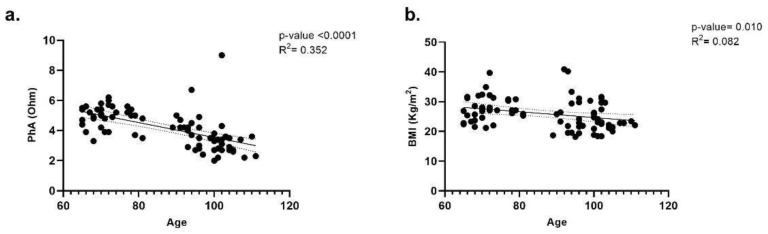
Correlation between age and PhA (**a**) and BMI (**b**). Linear regression analysis shows the relationship between PhA and age (**a**) in N = 80 individuals. Each point represents data from each individual. The dashed line represents the 95% confidence interval, while the solid line indicates the data trend. PhA = Phase Angle; BMI = Body Mass Index; R^2^ = R squared.

Analyzing the correlation between PhA, BMI and age, the negative slope highlights a decline in PhA and BMI with increasing age (Figure 3).

However, the analysis is more robust for PhA than BMI (Figure 3a), as the R^2^ for BMI is close to zero, indicating an almost negligible correlation between BMI and age (Figure 3b). While the correlation between age and the anthropometric parameters is statistically significant in both cases, it holds meaningful relevance only for PhA, where a clear statistical association with age can be observed.

Finally, BIVA was conducted. It showed that the 65–80 age group has the lowest values for Rz (Figure S2), indicating relatively better hydration compared to older age groups (Figure S2). The ≥95 age group had the highest and lowest values of Rz and Xc, suggesting reduced hydration and body cell mass, which are typical in advanced aging (Figure S2). The ellipses overlap indicates the variability between the three groups, confirmed by the statistical analysis performed by BIVA software, which showed that the main difference between groups is for those aged 65–80 vs. ≥95 (Hotelling test, T^2^ = 56; F = 27.6; *p*-value = 0.0000; D = 1.8), followed by the 65–80 vs. 81–94 groups (T^2^ = 9.5; F = 4.7; *p*-value = 0.0146; D = 1.01) and the 81–94 vs. ≥95 groups (T^2^ = 7.4; F = 3.6; *p*-value = 0.0346; D = 0.88).

This interpretation is confirmed by the analysis of Rz and Xc normalized for height (Figure S3).

### 3.2. Nutritional Status Evaluation Based on MNA Questionnaire and CONUT and GNRI Scores

Table 2 presents the percentages of individuals belonging to each analyzed age group across the classifications for the scores and indices considered in the study.

The MNA evaluation showed that 69% of the 81–94 age group and 65% of the ≥95 age group were identified as being at risk of malnutrition (see Table 2). Only 8% and 9% of individuals in these respective age groups were classified as malnourished, whereas none of the youngest individuals in the cohort (65–80 years old) exhibited signs of malnutrition (Table 2). Additionally, no individuals were classified as undernourished regardless of group affiliation. The GNRI and CONUT scores exhibited a similar distribution across age groups. From the age of 80 onward, the GNRI distribution was concentrated in the “no risk” and “low risk” categories, while the CONUT score was primarily distributed between the “normal” and “light” assessment categories (Table 2). In the youngest age group (64–80), all individuals were classified as “no risk” according to GNRI, while 82% fell within the “normal” assessment category for CONUT. In the LLIs group, the percentages were evenly distributed across the three categories for both GNRI and CONUT (Table 2).

A negative correlation between the parameters of GNRI (i.e., albumin and ideal weight) and age was found, confirming the data about the reduced levels of albumin with increasing age (see Table S2).

## 4. Discussion

According to the European Society for Clinical Nutrition and Metabolism (ESPEN) guidelines (2002) for adult nutrition screening, as outlined in the Malnutrition Universal Screening Tool (MUST), malnutrition is assessed based on the following criteria: BMI < 20 kg/m^2^, unintentional weight loss greater than 5% within the last 3 months, reduced dietary intake (below 50–75% of usual requirements) for more than one week, and the presence of serious illness or major surgery, which can significantly increase nutritional assessment requirements [27]. Establishing this status is particularly critical upon hospital admission, as it helps identify patients at risk of malnutrition-related complications and guides appropriate clinical management [28].

Regarding the tools used to assess nutrition-related risks, such as malnutrition, in the older population (ages 65 to 95), BMI and the MNA questionnaire, which includes the assessment of BMI, are the most used, as they are considered well suited to address the changes associated with aging, including in hospital settings [14,29].

Moreover, according to this parameter, values higher than 29.9 kg/m^2^ indicate obesity, often associated with the onset of related inflammatory diseases. However, it must be considered that the older population is subject to the “obesity paradox” (e.g., older adults who are overweight or obese may experience better health outcomes than those with normal weight or those who are underweight), which challenges the validity of BMI as a health measure in these individuals due to changes in body composition, such as spinal curvature, loss of lean muscle mass, and redistribution of fat [30,31,32,33]. Despite this consideration, BMI remains one of the most widely used measures, even in the assessment of nutritional risk during hospitalization of individuals with comorbidities. In some cases, an inverse association has been observed between BMI value and the risk of malnutrition in older adults, as demonstrated in a study of hospitalized individuals with a mean age of 82.0 ± 11.4 years [29].

Besides BMI, BIA is also considered an accurate method for assessing nutritional and health status in the general population. It is based on the calculation of PhA, a parameter derived from Xc and Rz. PhA serves as an indicator of cell membrane integrity, muscle quality, and overall physiological resilience, all of which are closely related to body fluid distribution. However, this evaluation changes when the analyzed populations are the older ones and, in particular, the LLIs because, in that case, other factors have to be taken into account, and the nutritional assessment, from a clinical point of view, becomes more complicated. Indeed, in LLIs, age-related changes such as altered body water distribution, reduced total body water, increased edema formation, and shifts in fat distribution compromise the reliability of PhA as an accurate assessment tool for this population [5,34]. Furthermore, there is a lack of standardization for PhA in individuals aged 85 and older. Most studies and guidelines for BIA and PhA were conducted in younger populations, and applying these measures to LLIs or centenarians can be problematic due to the unique physiology and health challenges of these age groups. Interestingly, our findings confirmed these limitations: the majority of individuals over 95 years old were found to have a normal BMI (i.e., 68%, Table 1), comparable to weight ranges reported in a study of Portuguese centenarians [35]. However, these data were not in line with the BIA and BIVA results, which placed most LLIs in the quadrant of the cachectic state (i.e., PhA = 3–4°, Figure 1e,f and Figure S2). Thus, the second paradox that emerged from our analysis concerns an observational finding. Although lower PhA values are associated with poorer health conditions that reflect cell death or a breakdown in the selective permeability of the cell membrane (i.e., edema) [36], most of the older individuals recruited through home visits did not exhibit phenotypic characteristics typical of cachectic ones. Furthermore, from a clinical perspective, they did not show clear risk factors indicative of severely compromised health, aside from those commonly associated with advanced age, confirming that this age group represents a distinct subset of exceptionally resilient individuals, as shown in other studies on LLIs [18,37,38].

BIVA confirmed the data observed with BIA methods while highlighting the substantial variability in body composition across different age groups. Notably, the distinct separation of the ≥95 age group between the ellipse of BIVA (Figures S2 and S3) suggests a significant shift in body composition with very advanced age. BIVA, like BIA, is influenced by age-related changes in fluid distribution and may be prone to inaccuracies, particularly in individuals with edema or significant muscle loss, which can distort impedance measurements. It is also due to the limitation of the method used (single-frequency), which does not permit the individuation of the exact distribution of fluids and lean mass in the body, with a loss of accuracy in distinguishing extracellular from intracellular water [39].

In addition to impedance-based techniques, we explored other nutritional screening tools such as MNA, CONUT, and GNRI, which are widely used to identify nutritional risk and its impact on ARDs [17,30,40,41,42,43]. While MNA is validated for individuals aged ≥ 65, its applicability in LLIs has been explored less, as most studies focus on the impact of past dietary habits on longevity rather than real-time nutritional assessments. On the other hand, GNRI and CONUT scores offer a quick and effective way to assess nutritional health risk, taking advantage of biochemical parameters that are easily accessible even for the older population [17,42,43]. Studies have shown a strong link between high GNRI risk and low albumin levels, both associated with higher risks of hypertension, cardiovascular diseases, and overall mortality because a reduction in serum albumin could be a sign of an increased inflammatory status instead of undernutrition conditions [25,44,45,46,47]. This is also highlighted in a study evaluating the nutrition status of hospitalized older adults using the MUST and Global Leadership Initiative on malnutrition criteria (GLIM). The findings revealed a lack of correspondence between the rate of malnutrition and albumin levels in the group classified as being at lower nutritional risk but with inflammatory status, suggesting a possible mismatch between clinical classification and biochemical markers [29]. Moreover, GNRI has been recognized as a prognostic indicator for length of hospital stay and chronic conditions in older patients [48]. In the same way, CONUT integrated some biochemical parameters, offering a comprehensive look at nutritional status, including protein reserves (i.e., serum albumin), caloric intake (i.e., total cholesterol), and immune function (i.e., lymphocyte count) [46]. Furthermore, higher CONUT scores have been associated with poorer clinical outcomes, such as higher rates of infection, complications, and mortality in hospitalized patients, permitting identification of those who may benefit from nutritional support [49].

Considering the two parameters of GNRI (i.e., albumin levels and body weight) individually, we found that only 38% of the LLIs in our cohort had albumin levels below the normal threshold, which typically indicates an inflammatory state. Interestingly, the majority of them had albumin levels above the normal reference range, suggesting that, despite their advanced age, their nutritional status was relatively well maintained. This observation is encouraging, as it implies that these individuals had preserved their nutritional health to a certain extent, even as they aged, and once again, it contrasts with the data regarding PhA and the cachectic state. Consequently, most of the LLIs in our cohort were classified in the moderate or low-risk categories according to the GNRI score (i.e., 3% and 44%, respectively), indicating a generally good nutritional status (Table 2).

The data from CONUT suggest a trend of declining nutritional status from the youngest to the oldest individuals considered in the study (Table 2), evidenced by the decrease in the normal category and the rise in light and moderate malnutrition. The potential cause for this could reflect a kind of worsening of health conditions according to age groups and their impact on nutritional status. In the youngest age group (65–80), 82% fell within the “normal” CONUT category, indicating overall good nutritional status. From the age of 80 onward, the CONUT score was mostly distributed between the “normal” and “light” categories. However, in the oldest group (i.e., ≥95), the distribution across all three categories suggests increased heterogeneity in nutritional status with advancing age.

On MNA, our data provide valuable insights into the nutritional status of older individuals, revealing clear patterns across different age groups (Table 2). The data obtained in the 65–80 years old group (mostly well-nourished, 88%) may suggest that, up to the age of 80, the majority of older adults can maintain good nutritional status despite the typical challenges associated with aging. Factors like access to adequate food and fewer functional impairments could contribute to this relatively healthy state of nutrition [17,50]. If previous studies have shown that only 12.3% of a large sample of centenarians achieved good nutritional status [50], we observed that 23% of the cohort of LLIs were well fed (Table 2). Furthermore, despite the high proportion of individuals in these age groups who are at risk of malnutrition (i.e., 65%), the number of malnourished individuals was relatively low (i.e., 9%, Table 2). It is also worth noting that no individuals, regardless of age group, were classified as undernourished. The absence of undernourishment across all groups suggests that, while many older adults are at risk of malnutrition, they are not generally experiencing the most severe forms of nutritional deprivation. These data are quite close to those observed in a cohort of 139 centenarians in a Colombian study, in which the majority of them showed an elevated risk of malnutrition, followed by a 36% prevalence of malnourished individuals and 17% prevalence of well-fed individuals [51]. Despite these interesting findings, the primary limitation of the MNA is the integration with BMI values. However, the nutritional status assessed by the MNA is likely more accurate than that inferred from BMI, as age-related body composition changes can distort interpretations based on impedance-based measurements, as discussed by Guigoz et al. [40]. Additionally, the administration of nutritional questionnaires can be influenced by the living environment of aged people, introducing potential biases.

Several studies comparing the nutritional assessment tools analyzed in this paper have demonstrated differing approaches between the CONUT and MNA scores with regard to evaluating the nutritional status of older individuals. For example, Formiga et al. found that MNA tended to underestimate the nutritional status of hospitalized patients aged 85 and over when compared to CONUT, which more accurately identified those in better nutritional condition [52]. Accordingly, the CONUT score appears to function primarily as a marker of disease-related malnutrition, particularly in acute or hospital settings, where nutritional deterioration is often influenced by systemic inflammation. In our data, the inflammatory status, determined by albumin dosage and the anamnestic information, should not correlate with the nutritional status but could be a possible factor in the determination of heterogeneity in the CONUT score in LLIs. However, few studies have systematically examined and compared these scores specifically in older or very old populations, and the available evidence remains conflicting. For instance, Lo Buglio et al. reported an inverse correlation between MNA and CONUT, suggesting differing sensitivities to clinical and inflammatory changes [53]. Similarly, Uemura et al., in a study comparing MNA, CONUT, and GNRI in patients with acute heart failure, found that the three scores were correlated only in the subgroup with cardiovascular disease, indicating that the clinical context may significantly influence score alignment and predictive capacity [54].

In summary, nutritional risk and malnutrition increase with age, as indicated by MNA, GNRI, and CONUT scores. The 65–80 age group had the highest PhA values, which progressively decreased with age. This group also had the highest proportion of well-fed individuals (88%), with only 12% at risk of malnutrition and none classified as malnourished. All individuals in this group had no GNRI risk, and 82% were classified as having a normal nutritional status according to CONUT, while 18% exhibited light malnutrition, and none had moderate malnutrition. In contrast, LLIs (≥95 years) had the lowest PhA values and lower well-fed percentages, with a higher malnutrition risk according to the MNA, similar to the 81–94 age group. Both the 81–94 and ≥95 groups showed a decline in GNRI and CONUT assessments, both indices that are independent of anthropometric measurements, with light-to-moderate nutritional risk classifications, reinforcing the idea that nutritional risk increases with age. However, a substantial proportion of LLIs maintained a relatively stable nutritional status, suggesting a potential resilience among these individuals.

### Study Limitations

This study presents some limitations that must be acknowledged. No a priori sample size calculation was performed, as the cohort was based on the availability of eligible individuals, particularly LLIs (≥95 years), who represent a rare and underrepresented population in nutritional research. As a result, although the sample is valuable and unique, the relatively small group size in some age categories may limit the generalizability and statistical power of the findings. The cross-sectional design does not allow for the assessment of causal relationships between nutritional indices and clinical outcomes. Longitudinal studies would be required to better understand how changes in these indices over time relate to health trajectories in older populations. Furthermore, this study was conducted considering non-hospitalized individuals with a generally good health status and without chronic manifestations. To draw attention to the lack of clinical assessment of the older population, this study considered commonly used tools such as MNA, GNRI, CONUT, and BIA-derived PhA that were not originally designed or validated specifically for use in LLIs. Their applicability in this age group may be limited due to age-related physiological changes, such as altered body composition, inflammation, or hydration shifts, which may affect the accuracy of nutritional risk classification. This limitation permits us to underline the necessity of improving the standardization of a method that involves multidisciplinary assessment, but also the development of an age-specific approach to the evaluation of nutritional-related risk.

Moreover, some variables, such as dietary intake, physical activity, or comorbidities, were self-reported or extracted from medical records, potentially introducing recall bias or inconsistencies in clinical documentation. Other variables, such as medication use and ongoing treatments with specific drug classes, were not included in the correlation analysis of this study, representing a minor limitation. Nevertheless, these factors are clinically relevant and warrant further investigation in future research based on a more comprehensive dataset.

Finally, excluding individuals with severe cognitive decline may limit the applicability of our findings to advanced stages of dementia. Thus, future studies specifically targeting patients with pronounced cognitive impairment will expand the knowledge for older adults.

Despite these limitations, the study offers important insights into the nutritional status of very old individuals and highlights the need for more targeted, multidimensional tools for assessing nutrition-related risk in this growing population segment.

## 5. Conclusions

Our data indicate that nutritional status follows an age-dependent trajectory: most individuals aged 65–80 maintain adequate nutrition, but malnutrition risk rises notably in the 81–94 group and persists at high levels among those over 95. This aligns with our observation of a progressive decline in PhA with advancing age. However, lower PhA values in our cohort did not consistently correlate with severe malnutrition or cachexia, suggesting potential limitations in interpreting PhA as a standalone marker in the oldest old.

Notably, LLIs (≥95 years) exhibited stable nutritional profiles despite low PhA values, a finding that may reflect resilience mechanisms undetected by conventional assessment tools. These results underscore the complexity of evaluating nutrition in extreme age groups, particularly given that methods like MNA, GNRI, and CONUT were not originally validated for the oldest old. While CONUT and GNRI offer advantages (e.g., independence from anthropometric changes common in aging), their clinical utility in this population requires further verification.

Methodological limitations of this pilot study, including its cross-sectional design, modest sample size, and lack of adjustment for confounders (e.g., smoking, physical activity, comorbidities), necessitate cautious interpretation. Future research should prioritize longitudinal cohorts and multivariate models to disentangle age-specific risk factors and validate assessment tools across subgroups.

In summary, biochemical scores such as CONUT and GNRI may complement multidimensional frameworks like ESPEN and GLIM, but their integration into clinical practice for the oldest old demands rigorous validation. Clarifying their role in personalized nutritional interventions remains a critical challenge for promoting healthy longevity.

## 6. Future Perspectives

Based on current knowledge of the nutritional status of the older population, and, in particular, of LLIs, and on the existing tools already in use, there is a growing need, both clinically and in basic research, to strengthen the multidisciplinary and scientific approach toward developing standardized and unified methods for assessing and stratifying nutritional risk in the very old. The extreme heterogeneity of this population, both in terms of physiology and clinical presentation, requires the creation of flexible and adaptive criteria capable of reflecting the wide variability observed among individuals aged 95 years and older.

To address these challenges, future studies should aim to expand the study cohort and employ more robust and tailored statistical methods, which could serve as a foundation for identifying the most effective combination of available assessment tools, adjusting them for clinically relevant variables and basic ones as well (i.e., living and smoking habits, diseases, consumption of drugs). This would facilitate the development of validated models for risk classification and intervention planning, ensuring more accurate and personalized nutritional care for this unique and growing population segment.

Moreover, prospective longitudinal research is needed to explore how nutritional scores change over time and how they relate to functional decline, comorbidity burden, and mortality. Integrating biochemical markers, frailty indices, and functional status into comprehensive nutritional assessment frameworks may also contribute to a more holistic understanding of health in LLIs, as well as the integration with information with specific drug classes (e.g., diuretics).

Excluding individuals with severe cognitive decline may limit the applicability of our findings to advanced stages of dementia. Thus, future studies specifically targeting patients with pronounced cognitive impairment will expand the knowledge base for older adults.

Ultimately, these efforts will support the creation of specific screening protocols and intervention strategies capable of improving both clinical outcomes and quality of life in the oldest population.

## Figures and Tables

**Table 1:** Age-based classification of groups according to BMI categories and values.

	Age Groups			*p*-Value
	65–80 N = 33 F/M = 16/17	81–94 N = 13 F/M = 8/5	≥95 N = 34 F/M = 24/10	
BMI (kg/m^2^) ± SD	27.65 ± 4.2	26.93 ± 3.9	23.91 ± 4.02	0.006 (65–80 vs. ≥95)
Underweight (%)	0	0	6	
Normal weight (%)	27	38	68	
Overweight (%)	39	38	15	
Obesity (%)	33	8	12	
Extreme obesity (%)	0	15	0	

N = number of individuals for each age; F = females; M = males; BMI = Body Mass Index; SD = standard deviation.

**Table 2:** Classification of age groups according to MNA, GNRI, and CONUT classes.

Age Groups			
	65–80 N = 33 F/M = 16/17	81–94 N = 13 F/M = 8/5	≥95 N = 34 F/M = 24/10
**MNA**			
Well fed (%)	88	23	26
At risk of malnutrition (%)	12	69	65
Malnourished (%)	0	8	9
**GNRI**			
No risk (%)	100	62	53
Moderate risk (%)	0	0	3
Low risk (%)	0	38	44
**CONUT**			
Normal (%)	82	61.5	53
Moderate (%)	0	7.5	3
Light (%)	18	31	44

N = number of individuals for each age group; F = females; M = males; MNA = Mini Nutritional Assessment; GNRI = Geriatric Nutritional Risk Index; CONUT = Controlling Nutritional status.

## Data Availability

The data presented in this study are available on request from the corresponding author due to privacy restrictions.

## Supplementary Materials

The following supporting information can be downloaded at https://www.mdpi.com/article/10.3390/nu17111873/s1, Table S1: Reference ranges for nutritional status based on PhA assessment; Table S2. Nutritional-related biomarkers (serum albumin, cholesterol, creatinine, C-reactive protein, HDL, LDL, white blood cells and lymphocytes) values according to age-based groups; Figure S1: Example of BIA ellipse with types of classification; Figure S2: BIVA confidence ellipse according to age groups; Figure S3: Rz and Xc normalized for height.

## Abbreviations

The following abbreviations are used in this manuscript:

BIA	Bioimpedance analysis.
MNA	Mini Nutritional Assessment.
CONUT	Controlling Nutritional Risk Index.
GNRI	Geriatric Nutritional Risk Index.
ARDs	Age-Related Diseases.
DRM	Disease-related malnutrition.
LLIs	Long-living individuals.
y.o.	Years old.
BMI	Body Mass Index.
STROBE	Strengthening the Reporting of Observational Studies in Epidemiology.
SD	Standard deviation.
MMSE	Mini Mental State Examination.
BIVA	Bioelectrical Impedance Vector Analysis.
PhA	Phase Angle.
Rz	Reactance.
Xc	Resistance.
H	Height.
WLo	Ideal Weight.
N	Number of individuals for each age.
F	Females.
M	Males.
ESPEN	European Society for Clinical Nutrition and Metabolism.
MUST	Malnutrition Universal Screening Tool.
GLIM	Global Leadership Initiative on malnutrition criteria.
