# Supplementary material for: Refining Nutritional Assessment Methods for Older Adults: A Pilot Study on Sicilian Long-Living Individuals
Source: Nutrients. 2025 May 30;17(11):1873. doi: 10.3390/nu17111873 (PMC12156965; doi:10.3390/nu17111873)
Supplement: Supplementary file 1 [file nutrients-17-01873-s001.zip › nutrients-3644912-supplementary.pdf]

nutrients

Supplementary file

Table S1. Reference ranges for nutritional status based on PhA assessment.

PhA (°)	Interpretation	References
< 3°	Severe pathological significance	[40-42]
< 6°	Poor prognosis, potential health complications	[43]
3° - 4°	Diseases / strong catabolic state/edema	[40-42]
4° - 5.5°	Medium catabolic state/water retention	[40-42]
≥ 6.96°	Healthy individuals	[43]
> 7.5°	Tendency to dehydration in average or robust individuals	[40-42]
> 8°	Typical values in athletes	[43]

PhA = Phase Angle

Table S2. Nutritional-related biomarkers (serum albumin, cholesterol, creatinine, C-reactive protein, HDL, LDL, white blood cells and lymphocytes) values according to age-based groups.

	Age groups			Significance	p-value
	65-80<br>N = 33<br>F/M = 16/17	81-94<br>N = 13<br>F/M = 8/5	≥ 95<br>N = 34<br>F/M = 24/10		
Albumin (g/L) ± SD	43.89 ± 3.11	40.04 ± 2.27	38.81 ± 3.7	65-80 *vs.* 81-94	0.002
				81-94 *vs.* ≥ 95	<0.0001
Cholesterol (mg/dL) ± SD	189 ± 27.76	166.2 ± 36.01	168.4 ± 31.52	65-80 *vs.* 81-94	0.04
				65-80 *vs.* ≥ 95	0.009
Creatinine (mg/dL) ± SD	0.85 ± 0.22	1.11 ± 0.36	1.19 ± 0.55	65-80 *vs.* ≥ 95	0.001
CRP (mg/dL)	3.46 ± 5.62	12.18 ± 16.14	4.92 ± 5.95	65-80 *vs.* 81-94	0.0038
				81-94 *vs.* ≥ 95	0.015
HDL (mg/dL) ± SD	55.43 ± 10.21	44.80 ± 7.5	55.45 ±13.68	65-80 *vs.* 81-94	0.014
				81-94 *vs.* ≥ 95	0.014
LDL (mg/dL)	135.3 ± 121.8	95.97 ± 33.97	94.06 ± 27.09	ns	ns
WBC (10^ 3/µL)	6.57 ± 1.87	6.56 ± 1.61	7 ± 1.9	ns	ns
Lymphocytes (10^ 3/µL)	2.11 ± 0.98	1.79 ± 0.92	1.9 ± 0.69	ns	ns

N = number of individuals for each age; F = females; M = males; SD = standard deviation; CRP = C-reactive protein; HDL = high density lipoprotein; LDL = low density lipoprotein; WBC = white blood cells; ns = not significant; $p < .05$ was not considered significant.

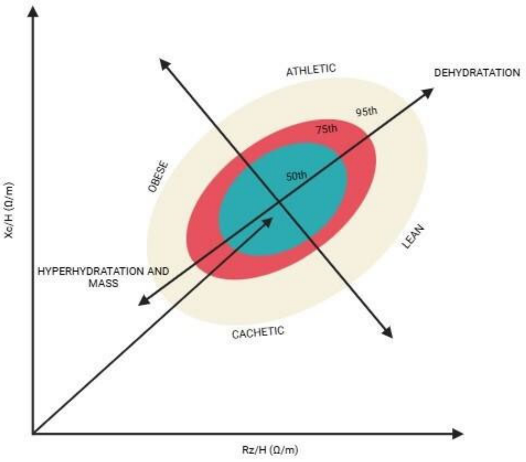

Figure S1. Example of BIA ellipse with types of classification. Vector shifts in the nomogram indicate hydration and tissue mass changes: upward shifts suggest dehydration, while downward shifts indicate hyperhydration. Leftward movement corresponds to increased tissue hydration and mass, whereas rightward movement reflects their reduction. Healthy individuals typically fall in the upper-left quadrant ("athletic" profile), while dehydration aligns with the upper-right, increased tissue hydration with the lower-left, and cachexia with the lower-right. Percentiles (50th, 75th, 95th) aid body composition comparisons [44, 45].

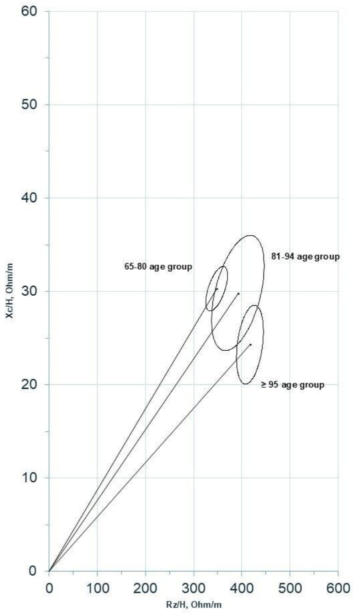

Figure S2. BIVA confidence ellipse according to age groups. Rz/H = Resistance/Height; Xc/H = Reactance/Height.

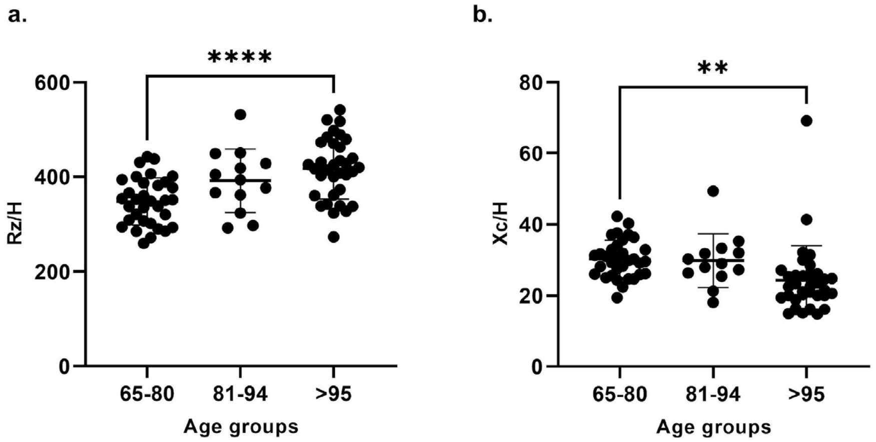

Figure S3. Rz and Xc normalized for height. The scatter plot shows differences between the mean of the values of Rz/H (**a**) and Xc/H (**b**) from each age group obtained by one-way ANOVA test. The SD and p-values are shown on the graphs. The vertical lines with horizontal caps represent the mean ± SD. Statistical significance between groups in the columns is denoted by horizontal lines above the bars, marked with asterisks (*). The number of "*" indicates the level of significance: ** p-value ≤ 0.01; ****p-value ≤ 0.0001. Rz/H = Resistance/Height; Xc/H = Reactance/Height.
